# Cerebrospinal fluid volume improves prediction of malignant edema after endovascular treatment of stroke

**DOI:** 10.1177/17474930221094693

**Published:** 2022-05-12

**Authors:** Frans Kauw, Marie Louise E Bernsen, Jan W Dankbaar, Hugo WAM de Jong, L Jaap Kappelle, Birgitta K Velthuis, H Bart van der Worp, Aad van der Lugt, Yvo BWEM Roos, Lonneke SF Yo, Marianne AA van Walderveen, Jeannette Hofmeijer, Edwin Bennink

**Affiliations:** 1 Department of Radiology, University Medical Center Utrecht, Utrecht University, Utrecht, The Netherlands; 2 Department of Neurology and Neurosurgery, Brain Center, University Medical Center Utrecht, Utrecht University, Utrecht, The Netherlands; 3 Department of Radiology, Rijnstate Hospital, Arnhem, The Netherlands; 4 Department of Radiology, Erasmus MC, University Medical Center, Rotterdam, The Netherlands; 5 Department of Neurology, Amsterdam University Medical Center, Amsterdam, The Netherlands; 6 Department of Radiology, Catharina Hospital, Eindhoven, The Netherlands; 7 Department of Radiology, Leiden University Medical Center, Leiden, The Netherlands; 8 Department of Neurology, Rijnstate Hospital, Arnhem, The Netherlands; 9 Image Sciences Institute, University Medical Center Utrecht, Utrecht University, Utrecht, The Netherlands

**Keywords:** Ischemic stroke, computed tomography, cerebrospinal fluid, thrombectomy, malignant edema

## Abstract

**Background:**

The ratio of intracranial cerebrospinal fluid (CSF) volume to intracranial volume (ICV) has been identified as a potential predictor of malignant edema formation in patients with acute ischemic stroke.

**Aims:**

We aimed to evaluate the added value of the CSF/ICV ratio in a model to predict malignant edema formation in patients who underwent endovascular treatment.

**Methods:**

We included patients from the MR CLEAN Registry, a prospective national multicenter registry of patients who were treated with endovascular treatment between 2014 and 2017 because of acute ischemic stroke caused by large vessel occlusion. The CSF/ICV ratio was automatically measured on baseline thin-slice noncontrast CT. The primary outcome was the occurrence of malignant edema based on clinical and imaging features. The basic model included the following predictors: age, National Institutes of Health Stroke Scale, Alberta Stroke Program Early CT score, occlusion of the internal carotid artery, collateral score, time between symptom onset and groin puncture, and unsuccessful reperfusion. The extended model included the basic model and the CSF/ICV ratio. The performance of the basic and the extended model was compared with the likelihood ratio test.

**Results:**

Malignant edema occurred in 40 (6%) of 683 patients. In the extended model, a lower CSF/ICV ratio was associated with the occurrence of malignant edema (odds ratio (OR) per percentage point, 1.2; 95% confidence interval (CI) 1.1–1.3, *p* < 0.001). Age lost predictive value for malignant edema in the extended model (OR 1.1; 95% CI 0.9–1.5, *p* = 0.372). The performance of the extended model was higher than that of the basic model (*p* < 0.001).

**Conclusions:**

Adding the CSF/ICV ratio improves a multimodal prediction model for the occurrence of malignant edema after endovascular treatment.

## Introduction

The occurrence of malignant edema may require timely decompressive surgery to prevent poor clinical outcomes in patients with acute ischemic stroke.^1,2^ Accurate prediction of malignant edema formation may help in making prompt treatment decisions. Important factors that have been associated with malignant edema include young age, high National Institutes of Health Stroke Scale (NIHSS), extensive early ischemic changes on noncontrast CT (NCCT), large perfusion deficits on CT perfusion, proximal thrombus location on CT angiography (CTA), and poor collateral filling in the affected area on CTA.^3^ The incorporation of these factors in multivariable models has led to reasonable discrimination between groups of patients with and without malignant edema, but predictive values for individual patients remain moderate.^3^

In a previous study, we found the ratio of intracranial cerebrospinal fluid (CSF) volume to intracranial volume (ICV) to be of added value in models predicting malignant edema in patients with acute ischemic stroke.^4^ Until now, this has not been validated in another cohort.

Recently, a prediction model for malignant edema after acute supratentorial ischemic stroke for which endovascular treatment (EVT) was performed has been developed based on the Multicenter Randomized Controlled Trial of Endovascular Treatment for Acute Ischaemic Stroke in the Netherlands (MR CLEAN) Registry.^5^ In this study, we tested the additional predictive value of the CSF/ICV ratio.

## Methods

### Study design

Patients were selected from the MR CLEAN Registry.^6^ All patients undergoing EVT in the Netherlands were registered in this prospective national multicenter registry between 16 March 2014 and 1 November 2017. This study was reported according to The REporting of studies Conducted using Observational Routinely collected health Data (RECORD) Statement (See Supplemental Material).^7^

### Patient selection

**Figure 1.**
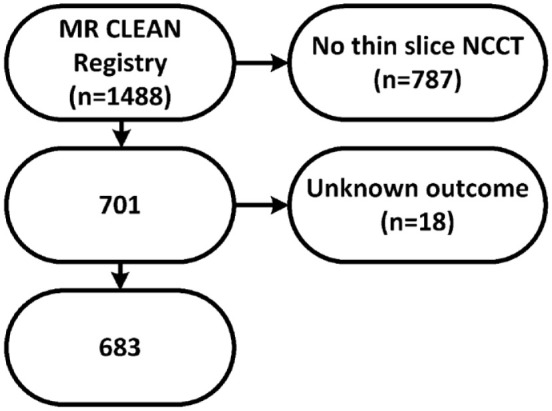
Flowchart of patient selection. NCCT: noncontrast CT.

Inclusion criteria were age ⩾ 18 years; the clinical diagnosis of acute ischemic stroke due to large vessel occlusion of the anterior circulation (intracranial carotid artery, M1, M2, A1, or A2) visualized with CTA; EVT initiated within 6.5 h of symptom onset or last seen well; and available thin-slice NCCT data on admission. As a result, 701 patients were included in our analysis (Figure 1).

### Ethics approval and data availability

The study protocol of the MR CLEAN Registry was approved by the central medical ethics committee of the Erasmus Medical Center Rotterdam, The Netherlands. The requirement for written informed consent was waived, but patients or their representatives were provided with information on the study and were given the opportunity to refuse participation. Source data will not be made available, since no patient approval was obtained for sharing anonymized data. However, detailed analytic methods and study materials will be made available to other researchers upon reasonable request to the corresponding author.

### Data collection

Baseline characteristics such as demographics, stroke characteristics, and imaging variables were available from the MR CLEAN Registry database. All patients underwent NCCT and CTA before EVT and digital subtraction angiography (DSA) as part of EVT. NCCT was performed after EVT in the case of neurological deterioration.

### Imaging analysis

Visual imaging assessments were performed independently by two of 21 core laboratory members (20 interventional neuroradiologists and one interventional neurologist). The observers were blinded to clinical information except for the side symptoms. NCCT, CTA, and DSA images were evaluated in separate sessions.

Early ischemic changes on NCCT were graded with the Alberta stroke program early CT score (ASPECTS).^8^

Intracranial occlusions were dichotomized into occlusion of the internal carotid artery (ICA) and occlusion of the middle cerebral artery (M1 or M2 segment) or anterior cerebral artery (A1 or A2 segment). Assessment of collateral filling on CTA was based on the extent of collateral filling in the territory of the occluded artery: 0 indicates 0% filling, 1 indicates > 0% and ⩽ 50% filling, 2 indicates > 50% and < 100% filling and 3 indicates 100% filling.^9^ Collateral scores were dichotomized as poor (scores of 0 or 1) or good (scores of 2 or 3).

Reperfusion after EVT was evaluated with the expanded thrombolysis in cerebral infarction (eTICI) scale on DSA.^10^ A score was assigned for the degree of reperfusion in the occluded area: an eTICI score of 0 reflects no reperfusion; 1 reflects minimal reperfusion, but no filling of the distal branches; 2A reflects 1–49%; 2B50 50–66%; 2B67 67–89%; 2C 90–99%; and 3 100% filling of the distal branches. Successful reperfusion was defined as an eTICI score of 2B50 or higher. If DSA images were only available in one plane then the highest possible eTICI score was 2A.

The ratio between intracranial CSF volume and ICV was automatically determined on thin-slice NCCT, as described previously.^4^ In short, the brain was coarsely segmented into three tissue regions (all intracranial CSF, gray matter, and white matter) by registering the ICBM 152 nonlinear atlas.^11–13^ Volume fractions were plotted against Hounsfield units resulting in a histogram for each coarse segmentation: one for gray matter, one for white matter, and one for CSF. Gaussian mixture models were subsequently fitted to these histograms. In this way, we acquired precise volume measures in noisy data without the need for precise segmentations. The areas under the three Gaussian mixture models combined reflect the ICV and the area under the CSF curve reflects the CSF volume.

### Outcome measures

The primary outcome was malignant edema, which was based on clinical and radiological criteria. Malignant edema was defined as (1) decompressive surgery or death within one week after stroke onset because of a clinical diagnosis of malignant edema or (2) clinical features of malignant edema (decreased consciousness, unilateral dilated pupil, and severe neurological deficit) together with a midline shift greater than 5 mm on NCCT. Based on the available data, patients were categorized into three outcome groups: malignant edema, no malignant edema or death with an unknown cause. Patients who were assigned to the category of death with unknown causes were excluded from the analysis (Figure 1).

The secondary outcome was functional outcome 90 days after stroke graded according to the modified Rankin Scale (mRS).

### Statistical analysis

Parametric and nonparametric tests, where appropriate, were used to compare the characteristics of the group with malignant edema and the group without.

For the prediction analyses, missing values were solved by using multiple imputations in SPSS version 24, as described previously.^5^

A previously developed multivariable model was used as the basic model, which included the following predictors: age, baseline NIHSS, ASPECTS, ICA occlusion, collateral score, time between symptom onset and groin puncture, and unsuccessful reperfusion (Table 1).^5^ An extended model was developed by adding the CSF/ICV ratio to the basic model. The c-statistics were compared with the DeLong test.^14^ The added value of the CSF/ICV ratio was assessed by comparing the goodness of fit of the two models using the likelihood ratio test. In addition, the predictive value of age and ASPECTS were evaluated in the extended model. The assumption of collinearity was not violated according to the calculated variance inflation factors. We report odds ratios (ORs), c-statistics, and 95% confidence intervals (CIs).

**Table 1.** Multivariable prediction models and the association with malignant middle cerebral artery infarction.

	Basic model^a^		Extended model^a^	
Predictor	OR (95% CI)	*p* value	OR (95% CI)	*p* value
Age (per 10 years younger)	1.5 (1.2–1.9)	0.002	1.1 (0.9–1.5)	0.372
Baseline NIHSS (per point higher)	1.1 (1.0–1.2)	0.027	1.1 (1.0–1.2)	0.039
ASPECTS (per point lower)	1.2 (1.0–1.4)	0.022	1.1 (1.0–1.3)	0.070
ICA occlusion	2.2 (1.1–4.6)	0.027	1.9 (0.9–4.0)	0.074
Collateral score (per point lower)	2.5 (1.6–4.2)	<0.001	2.8 (1.7–4.8)	<0.001
Onset to groin (per 60 min)	1.3 (1.0–1.8)	0.074	1.3 (0.9–1.7)	0.133
Unsuccessful reperfusion	1.6 (0.8–3.2)	0.198	1.9 (0.9–3.9)	0.087
CSF/ICV ratio (per percent lower)	–	–	1.2 (1.1–1.3)	<0.001
*C-statistic*	*0.856*		*0.876*	*0.141*
*Likelihood ratio test*				*<0.001*

ASPECTS: Alberta Stroke Program Early CT Score; CI: confidence interval; CSF: cerebrospinal fluid; ICA: internal carotid artery; ICV: intracranial volume; NIHSS: National Institutes of Health Stroke Scale; OR: odds ratio.

a Likelihood ratio test: *p* < 0.001.

## Results

Of the 701 patients, 18 died from an unknown cause and were excluded from the final analysis (Figure 1). Of the included 683 patients, 40 (6%) developed malignant edema. The mean age was 68 ± 14 years and 372 (54%) patients were male (Supplemental Table 1). One patient without malignant edema underwent decompressive surgery because of symptomatic intracranial hemorrhage. The median mRS at 90 days was higher in the group with malignant edema than in the group without malignant edema (median 6 versus 3, respectively, *p* < 0.001). The mean CSF/ICV ratio was lower in the group with malignant edema than in the group without malignant edema (9% ± 5% versus 14% ± 6%, respectively, *p* < 0.001). In the extended model (Table 1), a lower CSF/ICV ratio was associated with the occurrence of malignant edema (OR per percentage point, 1.2; 95% CI 1.1–1.3, *p* < 0.001). Age lost the predictive value for malignant edema in the extended model (OR 1.1; 95% CI 0.9–1.5, *p* = 0.372). Removing age from the extended model did not change the c-statistic of the model significantly (0.873). In addition, ASPECTS lost the predictive value in the extended model (OR 1.1; 95% CI 1.0–1.3, *p* = 0.070).

**Figure 2.**
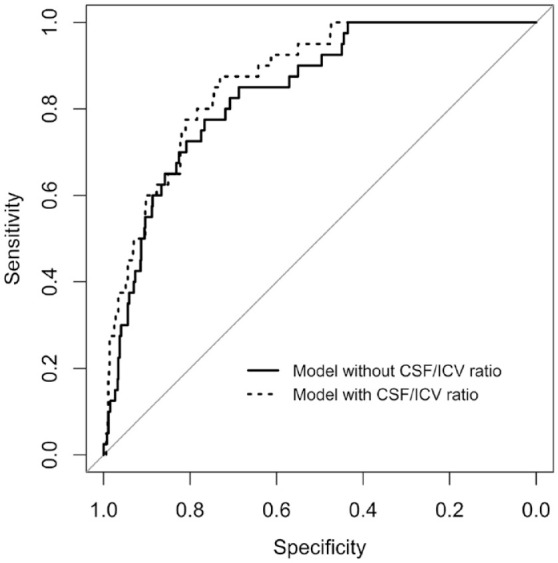
Receiver operating characteristic curves of the models with (dotted line) and without (solid line) the ratio of intracranial CSF volume to ICV. The area under the curve of the basic model (solid line) was 0.86 and the area under the curve of the extended model (dotted line) was 0.88 (*p* = 0.141). CSF: cerebrospinal fluid; ICV: intracranial volume.

The c-statistic of the extended model was slightly higher than the c-statistic of the basic model (0.88 versus 0.86, respectively, *p* = 0.141), which is also reflected in the ROC curves (Figure 2). The goodness of fit of the extended model was superior to the basic model (*p* < 0.001). An example case is shown in Supplemental Figure 1.

## Discussion

This study shows that in patients with ischemic stroke caused by large vessel occlusion, the CSF/ICV ratio on thin-slice NCCT is lower in patients who develop malignant edema after EVT than in those who do not, and that CSF/ICV ratio can improve a multimodal prediction model for malignant edema.

The predictive value of the CSF volume has been reported before.^4,15^ In a previous multicenter study, we showed the added value of the CSF/ICV ratio to three prediction models.^4^ In that study, we selected ischemic stroke patients independent of the treatment received. As a result, 70% received intravenous thrombolysis and 12% received endovascular treatment, whereas in the current study, we assessed only patients who received endovascular treatment. The high proportion of reperfusion in the current cohort may explain the relatively low incidence of malignant edema in this study (6%) compared to the previous study (12%). Another explanation may be the applied definition of malignant edema. In previous studies, a single imaging parameter (midline shift > 5 mm) was used, whereas in the current study, we use both clinical information and imaging features to determine the occurrence of malignant edema.^4,16^ Unfortunately, it was not feasible to perform a separate analysis with midline shift >5 mm as the outcome in this study. Despite these differences in methodology, the results regarding the added predictive value of the CSF/ICV ratio are similar.

The predictive value of age disappeared after the CSF/ICV ratio was added to our prediction model. In general, older patients have more CSF volume because of brain atrophy.^17^ In addition, older patients may have had previous brain disease more often than younger patients such as stroke, which also results in more intracranial space. Consequently, older patients have a larger “buffer” before brain swelling leads to midline shift or tissue herniation. In this study, previous ischemic stroke was more prevalent in the group without malignant edema than in the group with malignant edema. As age and the CSF/ICV ratio add similar predictive properties, one of the two predictors could be removed from the prediction model. Although age is readily available, and therefore more practical, the CSF/ICV ratio is a more direct reflection of the intracranial “buffer” and adds more to the prediction model. This is supported by studies that showed that older patients can still develop malignant edema.^18^

An interaction between the CSF/ICV ratio and ASPECTS has been proposed.^19^ Early signs of ischemia on NCCT reflect early swelling of the brain.^8^ As a result, it can be assumed that the CSF/ICV ratio is influenced by the degree of swelling in the acute phase. One might expect that the association between ASPECTS and malignant edema would weaken after adding the CSF/ICV ratio to the model, which was the case in this study. However, ASPECTS still had some predictive values in the extended model. The first possible explanation for this finding is that mixture model histograms were used to calculate the CSF and ICV volumes. This method is robust to noise, artifacts, and early ischemic changes such as swelling. The second possible explanation is that the time window between symptom onset and CT was short (6.5 h). The third possible explanation is that ASPECTS may involve only a (small) part of the brain, whereas the CSF/ICV ratio involves the whole brain.

Taken together with the previous study, in which the time window between symptom onset and CT was 9 h, we did not find evidence for an interaction between ASPECTS and the CSF/ICV ratio. Still, (automated) calculations of both ASPECTS and CSF/ICV can help to identify patients at risk of developing malignant edema.

Several models have been proposed for the prediction of malignant edema.^3^ Our analysis is in line with these studies with regard to the included predictors of malignant edema and the observed c-statistic of up to 0.88.^3^ The c-statistic (i.e. the area under the ROC curve) is often used to evaluate the discriminative performance of a prediction model, but it is not a sensitive measure for how well the model fits the data.^20^ Discrimination is only one aspect of model performance, whereas goodness-of-fit is a more global measure of model performance, which can be evaluated with the likelihood ratio test.^20^ This explains why the c-statistic of the extended model was slightly higher than the c-statistic of the basic model and the observed difference was insignificant, whereas the likelihood ratio test showed significant superiority of the extended model. This study is a step in finding the optimal prediction strategy for predicting malignant edema. Accurate prediction may lead to closer monitoring of patients at risk and timely decompressive surgery as warranted by the randomized controlled trials.^1^

Several strengths and limitations can be noted. A strength of this study was that a previously developed model, which was based on a cohort of 1445 patients, was used.^5^ Although variables such as ICA occlusion and unsuccessful reperfusion were not significant in the extended model due to a lack of power, we were able to reliably compare the basic model to the extended model, which was the primary aim of this study. In addition, the prospective nature of this multicenter study resulted in only a few missing values, which could be imputed. The second strength of this study was the determination of the CSF/ICV ratio, which was done automatically. The images can be analyzed within a few minutes and there is no risk of observer bias.

A limitation of this study was that we had to exclude many patients because no thin-slice data were available. In our opinion, the risk of selection bias is limited as we do not expect that the availability of thin-slice data is related to either predictors or outcomes. Nowadays, thin-slice NCCT data are available in almost every hospital in The Netherlands. In this study, all centers acquired thin-slice NCCT data, but not all the data were stored appropriately.

Another limitation of this study was the outcome assessment, which was done retrospectively. Although we believe that our definition of malignant edema was in line with other studies, we may have missed some cases. Another limitation was the lack of validation of the extended prediction model, which can be done in an external cohort.

In conclusion, adding the CSF/ICV ratio improves a multimodal prediction model for the occurrence of malignant edema after endovascular treatment.

## Supplemental Material

sj-pdf-2-wso-10.1177_17474930221094693 – Supplemental material for Cerebrospinal fluid volume improves prediction of malignant edema after endovascular treatment of stroke Click here for additional data file. Supplemental material, sj-pdf-2-wso-10.1177_17474930221094693 for Cerebrospinal fluid volume improves prediction of malignant edema after endovascular treatment of stroke by Frans Kauw, Marie Louise E Bernsen, Jan W Dankbaar, Hugo WAM de Jong, L Jaap Kappelle, Birgitta K Velthuis, H Bart van der Worp, Aad van der Lugt, Yvo BWEM Roos, Lonneke SF Yo, Marianne AA van Walderveen, Jeannette Hofmeijer and Edwin Bennink in International Journal of Stroke

sj-pdf-3-wso-10.1177_17474930221094693 – Supplemental material for Cerebrospinal fluid volume improves prediction of malignant edema after endovascular treatment of stroke Click here for additional data file. Supplemental material, sj-pdf-3-wso-10.1177_17474930221094693 for Cerebrospinal fluid volume improves prediction of malignant edema after endovascular treatment of stroke by Frans Kauw, Marie Louise E Bernsen, Jan W Dankbaar, Hugo WAM de Jong, L Jaap Kappelle, Birgitta K Velthuis, H Bart van der Worp, Aad van der Lugt, Yvo BWEM Roos, Lonneke SF Yo, Marianne AA van Walderveen, Jeannette Hofmeijer and Edwin Bennink in International Journal of Stroke

sj-pdf-4-wso-10.1177_17474930221094693 – Supplemental material for Cerebrospinal fluid volume improves prediction of malignant edema after endovascular treatment of stroke Click here for additional data file. Supplemental material, sj-pdf-4-wso-10.1177_17474930221094693 for Cerebrospinal fluid volume improves prediction of malignant edema after endovascular treatment of stroke by Frans Kauw, Marie Louise E Bernsen, Jan W Dankbaar, Hugo WAM de Jong, L Jaap Kappelle, Birgitta K Velthuis, H Bart van der Worp, Aad van der Lugt, Yvo BWEM Roos, Lonneke SF Yo, Marianne AA van Walderveen, Jeannette Hofmeijer and Edwin Bennink in International Journal of Stroke

sj-pdf-5-wso-10.1177_17474930221094693 – Supplemental material for Cerebrospinal fluid volume improves prediction of malignant edema after endovascular treatment of stroke Click here for additional data file. Supplemental material, sj-pdf-5-wso-10.1177_17474930221094693 for Cerebrospinal fluid volume improves prediction of malignant edema after endovascular treatment of stroke by Frans Kauw, Marie Louise E Bernsen, Jan W Dankbaar, Hugo WAM de Jong, L Jaap Kappelle, Birgitta K Velthuis, H Bart van der Worp, Aad van der Lugt, Yvo BWEM Roos, Lonneke SF Yo, Marianne AA van Walderveen, Jeannette Hofmeijer and Edwin Bennink in International Journal of Stroke

sj-tif-1-wso-10.1177_17474930221094693 – Supplemental material for Cerebrospinal fluid volume improves prediction of malignant edema after endovascular treatment of stroke Click here for additional data file. Supplemental material, sj-tif-1-wso-10.1177_17474930221094693 for Cerebrospinal fluid volume improves prediction of malignant edema after endovascular treatment of stroke by Frans Kauw, Marie Louise E Bernsen, Jan W Dankbaar, Hugo WAM de Jong, L Jaap Kappelle, Birgitta K Velthuis, H Bart van der Worp, Aad van der Lugt, Yvo BWEM Roos, Lonneke SF Yo, Marianne AA van Walderveen, Jeannette Hofmeijer and Edwin Bennink in International Journal of Stroke
