# Supplementary material for: Cerebrospinal fluid volume improves prediction of malignant edema after endovascular treatment of stroke
Source: Int J Stroke. 2022 May 12;18(2):187–92. doi: 10.1177/17474930221094693 (PMC9896253; doi:10.1177/17474930221094693)
Supplement: sj-pdf-2-wso-10.1177_17474930221094693 – Supplemental material for Cerebrospinal fluid volume improves prediction of malignant edema after endovascular treatment of stroke [file sj-pdf-2-wso-10.1177_17474930221094693.pdf]

Supplemental figure 1: Example case

Baseline (A) and follow-up non-contrast CT (B) of a 61-year-old male patient with an occlusion of the left internal carotid artery. Baseline NIHSS was 14. The intracranial cerebrospinal fluid (CSF) volume was 99 mL and the total intracranial volume (ICV) was 1504 mL resulting in a relatively small CSF/ICV ratio of 6.6 percent. The patient received intravenous thrombolysis and underwent endovascular treatment, but reperfusion was not achieved (eTICI 0). Almost five days after admission the patient developed malignant edema (B) and underwent hemicraniectomy. The clinical outcome after 3 months was poor with a modified Rankin Scale of 4.
