# Supplementary material for: Cerebrospinal fluid volume improves prediction of malignant edema after endovascular treatment of stroke
Source: Int J Stroke. 2022 May 12;18(2):187–92. doi: 10.1177/17474930221094693 (PMC9896253; doi:10.1177/17474930221094693)
Supplement: sj-pdf-3-wso-10.1177_17474930221094693 – Supplemental material for Cerebrospinal fluid volume improves prediction of malignant edema after endovascular treatment of stroke [file sj-pdf-3-wso-10.1177_17474930221094693.pdf]

Supplemental table 1. Patient characteristics

Characteristic, n (%)	Total n=683	No ME n=643	ME n=40	P value*
Age, mean±SD	68±14	69±14	62±13	0.004
Male sex	372 (54)	347 (54)	25 (62)	0.293
Pre-stroke mRS, median (Q1-Q3)	0 (0-1)	0 (0-1)	0 (0-0)	0.010
Current smoking	156 (23)	149 (23)	7 (17)	0.268
Admission NIHSS, median (Q1-Q3)	15 (11-20)	15 (11-19)	20 (16-22)	<0.001
Intravenous thrombolysis	539 (79)	506 (79)	33 (82)	0.579
**Medical history, n (%)**				
Hypertension	333 (49)	319 (50)	14 (36)	0.080
Diabetes mellitus	112 (17)	104 (16)	8 (20)	0.544
Hyperlipidemia	185 (28)	175 (28)	10 (26)	0.754
Ischemic stroke	102 (15)	100 (16)	2 (5)	0.083
Myocardial infarction	110 (16)	105 (17)	5 (13)	0.527
Atrial fibrillation	135 (20)	131 (21)	4 (10)	0.113
Peripheral arterial disease	50 (7)	50 (8)	0 (0)	0.071
**Drug use, n (%)**				
Antiplatelet	231 (34)	217 (34)	14 (35)	0.920
Coumarin	78 (12)	77 (12)	1 (2)	0.066
DOAC	15 (2)	14 (2)	1 (2)	0.902
Statin	231 (35)	221 (35)	10 (25)	0.189
**Imaging findings, n (%)**				
Occlusion site on CTA				<0.001
- Internal carotid artery	171 (25)	149 (23)	22 (55)	
- M1 segment of the MCA	392 (59)	376 (60)	16 (40)	
- M2 segment of the MCA	90 (14)	88 (14)	2 (5)	
- Other	10 (2)	10 (2)	0 (0)	
ASPECTS, median (Q1-Q3)	9 (7-10)	9 (7-10)	6 (5-9)	<0.001
Poor collateral score	276 (40)	245 (38)	31 (78)	<0.001
CSF volume, mL, mean±SD	171±78	174±77	112±61	<0.001
ICV, mL, mean±SD	1271±146	1274±146	1223±142	0.037
CSF/ICV percentage, mean±SD	13±6	14±6	9±5	<0.001
**Endovascular treatment, n (%)**				
Time from onset to groin, minutes, mean±SD	209±73	208±73	230±70	0.058
Duration of procedure, minutes, mean±SD	66±35	65±34	87±41	0.002
General anesthesia	249 (40)	228 (39)	21 (58)	0.020
eTICI 2B50-3	405 (60)	387 (61)	18 (46)	0.065
**Follow-up, n (%)**				
Decompressive surgery	15 (2)	1 (0)	14 (35)	<0.001
mRS at 90 days, median (Q1-Q3)	3 (2-6)	3 (2-5)	6 (4-6)	<0.001

* Either parametric or non-parametric tests were performed depending on the variable distribution.
ME indicates malignant edema; SD, standard deviation; mRS, modified Rankin Scale; Q1, first quartile; Q3, third quartile; NIHSS, National Institutes of Health Stroke Scale; CTA, computed tomography angiography; MCA, middle cerebral artery; ASPECTS: Alberta Stroke Program Early CT Score; NCCT: non-contrast computed tomography; CSF: cerebrospinal fluid; mL, milliliter; ICV, intracranial volume; eTICI, expanded thrombolysis in cerebral infarction.
