# Supplementary material for: Cerebrospinal fluid volume improves prediction of malignant edema after endovascular treatment of stroke
Source: Int J Stroke. 2022 May 12;18(2):187–92. doi: 10.1177/17474930221094693 (PMC9896253; doi:10.1177/17474930221094693)
Supplement: sj-pdf-4-wso-10.1177_17474930221094693 – Supplemental material for Cerebrospinal fluid volume improves prediction of malignant edema after endovascular treatment of stroke [file sj-pdf-4-wso-10.1177_17474930221094693.pdf]

Rotterdam, August 2019 - Registry part I+II

MR CLEAN Registry Investigators – group authors

Executive committee

Diederik W.J. Dippel[1];Aad van der Lugt[2];Charles B.L.M. Majoie[3];Yvo B.W.E.M. Roos[4];Robert J. van Oostenbrugge[5];Wim H. van Zwam[6];Jelis Boiten[14];Jan Albert Vos[8]

Study coordinators

Ivo G.H. Jansen[3];Maxim J.H.L. Mulder[1,2];Robert- Jan B. Goldhoorn[5,6];Kars C.J. Compagne[2];Manon Kappelhof[3];Josje Brouwer[4];Sanne J. den Hartog[1,2,40];Wouter H. Hinsenveld [5,6];

Local principal investigators

Diederik W.J. Dippel[1];Bob Roozenbeek[1];Aad van der Lugt[2];Adriaan C.G.M. van Es[2];Charles B.L.M. Majoie[3];Yvo B.W.E.M. Roos[4];Bart J. Emmer[3];Jonathan M. Coutinho[4];Wouter J. Schonewille[7];Jan Albert Vos[8]; Marieke J.H. Wermer[9];Marianne A.A. van Walderveen[10];Julie Staals[5];Robert J. van Oostenbrugge[5];Wim H. van Zwam[6];Jeannette Hofmeijer[11];Jasper M. Martens[12];Geert J. Lycklama à Nijeholt[13];Jelis Boiten[14];Sebastiaan F. de Bruijn[15];Lukas C. van Dijk[16];H. Bart van der Worp[17];Rob H. Lo[18];Ewoud J. van Dijk[19];Hieronymus D. Boogaarts[20];J. de Vries[22];Paul L.M. de Kort[21]; Julia van Tuijl[21] ; Jo P. Peluso[26];Puck Fransen[22];Jan S.P. van den Berg[22];Boudewijn A.A.M. van Hasselt[23];Leo A.M. Aerden[24];René J. Dallinga[25];Maarten Uyttenboogaart[28];Omid Eschgi[29];Reinoud P.H. Bokkers[29];Tobien H.C.M.L. Schreuder[30];Roel J.J. Heijboer[31];Koos Keizer[32];Lonneke S.F. Yo[33];Heleen M. den Hertog[22];Tomas Bulut[35]; Paul J.A.M. Brouwers[34]

Imaging assessment committee

Charles B.L.M. Majoie[3](chair);Wim H. van Zwam[6];Aad van der Lugt[2];Geert J. Lycklama à Nijeholt[13];Marianne A.A. van Walderveen[10];Marieke E.S. Sprengers[3];Sjoerd F.M. Jenniskens[27];René van den Berg[3];Albert J. Yoo[38];Ludo F.M. Beenen[3];Alida A. Postma[6];Stefan D. Roosendaal[3];Bas F.W. van der Kallen[13];Ido R. van den Wijngaard[13];Adriaan C.G.M. van Es[2];Bart J. Emmer[,3];Jasper M. Martens[12]; Lonneke S.F. Yo[33];Jan Albert Vos[8]; Joost Bot[36], Pieter-Jan van Doormaal[2]; Anton Meijer[27];Elyas Ghariq[13]; Reinoud P.H. Bokkers[29];Marc P. van Proosdij[37];G. Menno Krietemeijer[33];Jo P. Peluso[26];Hieronymus D. Boogaarts[20];Rob Lo[18];Wouter Dinkelaar[2]Auke P.A. Appelman[29];Bas Hammer[16];Sjoert Pegge[27];Anouk van der Hoorn[29];Saman Vinke[20].

Writing committee

Diederik W.J. Dippel[1](chair);Aad van der Lugt[2];Charles B.L.M. Majoie[3];Yvo B.W.E.M. Roos[4];Robert J. van Oostenbrugge[5];Wim H. van Zwam[6];Geert J. Lycklama à Nijeholt[13];Jelis Boiten[14];Jan Albert Vos[8];Wouter J. Schonewille[7];Jeannette Hofmeijer[11];Jasper M. Martens[12];H. Bart van der Worp[17];Rob H. Lo[18]

Adverse event committee

Robert J. van Oostenbrugge[5](chair);Jeannette Hofmeijer[11];H. Zwenneke Flach[23]

Trial methodologist

Hester F. Lingsma[40]

Research nurses / local trial coordinators

Naziha el Ghannouti[1];Martin Sterrenberg[1];Wilma Pellikaan[7];Rita Sprengers[4];Marjan Elfrink[11];Michelle Simons[11];Marjolein Vossers[12];Joke de Meris[14];Tamara Vermeulen[14];Annet Geerlings[19];Gina van Vemde[22];Tiny Simons[30];Gert Messchendorp[28];Nynke Nicolaij[28];Hester Bongenaar[32];Karin Bodde[24];Sandra Kleijn[34];Jasmijn Lodico[34]; Hanneke Droste[34];Maureen Wollaert[5];Sabrina Verheesen[5];D. Jeurrissen[5];Erna Bos[9];Yvonne Drabbe[15];Michelle Sandiman[15];Nicoline Aaldering[11];Berber Zweedijk[17];Jocova Vervoort[21];Eva Ponjee[22];Sharon Romviel[19];Karin Kanselaar[19];Denn Barning[10].

PhD / Medical students:

Esmee Venema[40]; Vicky Chalos[1,40]; Ralph R. Geuskens[3]; Tim van Straaten[19];Saliha Ergezen[1]; Roger R.M. Harmsma[1]; Daan Muijres[1]; Anouk de Jong[1];Olvert A. Berkhemer[1,3,6];Anna M.M. Boers[3,39]; J. Huguet[3];P.F.C. Groot[3];Marieke A. Mens[3];Katinka R. van Kranendonk[3];Kilian M. Treurniet[3];Manon L. Tolhuisen[3,39];Heitor Alves[3];Annick J. Weterings[3],Eleonora L.F. Kirkels[3],Eva J.H.F. Voogd[11];Lieve M. Schupp[3];Sabine L. Collette[28,29];Adrien E.D. Groot[4];Natalie E. LeCouffe[4];Praneeta R. Konduri[39];Haryadi Prasetya[39];Nerea Arrarte-Terreros[39];Lucas A. Ramos[39].

List of affiliations

Department of Neurology[1], Radiology[2], Public Health[40], Erasmus MC University Medical Center;
Department of Radiology and Nuclear Medicine[3], Neurology[4], Biomedical Engineering & Physics[39], Amsterdam UMC, University of Amsterdam, Amsterdam;
Department of Neurology[5], Radiology[6], Maastricht University Medical Center and Cardiovascular Research Institute Maastricht (CARIM);
Department of Neurology[7], Radiology[8], Sint Antonius Hospital, Nieuwegein;

Department of Neurology[9], Radiology[10], Leiden University Medical Center;
Department of Neurology[11], Radiology[12], Rijnstate Hospital, Arnhem;
Department of Radiology[13], Neurology[14], Haaglanden MC, the Hague;
Department of Neurology[15], Radiology[16], HAGA Hospital, the Hague;
Department of Neurology[17], Radiology[18], University Medical Center Utrecht;
Department of Neurology[19], Neurosurgery[20], Radiology[27], Radboud University Medical Center, Nijmegen;
Department of Neurology[21], Radiology[26], Elisabeth-TweeSteden ziekenhuis, Tilburg;
Department of Neurology[22], Radiology[23], Isala Klinieken, Zwolle;
Department of Neurology[24], Radiology[25], Reinier de Graaf Gasthuis, Delft;
Department of Neurology[28], Radiology[29], University Medical Center Groningen;
Department of Neurology[30], Radiology[31], Atrium Medical Center, Heerlen;
Department of Neurology[32], Radiology[33], Catharina Hospital, Eindhoven;
Department of Neurology[34], Radiology[35], Medisch Spectrum Twente, Enschede;
Department of Radiology[36], Amsterdam UMC, Vrije Universiteit van Amsterdam, Amsterdam;
Department of Radiology[37], Noordwest Ziekenhuisgroep, Alkmaar;
Department of Radiology[38], Texas Stroke Institute, Texas, United States of America.
